# Vaccinal Syphilis

**Published:** 1882-02-20

**Authors:** 


**Vaccinal Syphilis.**—A letter to La France Medicale (July 30, 1881) says that the Algerian journals are full of the most lamentable details regarding the numerous cases of syphilis which have appeared in the garrison of Algiers, following a public vaccination made on certain Algerian soldiers. It is said that fifty-eight young men have contracted syphilis by being vaccinated with lymph given by a syphilitic infant.—[Medical Times.

